# Are Online Synchronous Team-Based-Learning (TBL) pedagogy effective?: Perspectives from a study on medical students in Oman

**DOI:** 10.30476/JAMP.2021.92361.1481

**Published:** 2022-01

**Authors:** MOHAN B SANNATHIMMAPPA, VINOD NAMBIAR, RAJEEV ARAVINDAKSHAN, ANIL KUMAR

**Affiliations:** 1 College of Medicine and Health Sciences, Department of Microbiology, National University of Science and Technology, Sohar Campus, Sultanate of Oman, Oman; 2 Department of community medicine, All India Institute of Medical Sciences, Mangalagiri, Andhra Pradesh, India; 3 Department of Anatomy, College of Medicine and Health Sciences, National University of Science and Technology, Sohar Campus, Oman

**Keywords:** Active learning, Cognition, Immunology, Medical education

## Abstract

**Introduction:**

Team-based learning (TBL) is a highly structured, instructive, and student-focused pedagogy used by medical educators to foster the students’ learning. The present study aimed to qualitatively explore the students’ perception of the effectiveness of online synchronous TBL pedagogical strategy in promoting learning outcomes.

**Methods:**

A cross-over interventional study was conducted on MD4 year medical students, using four modified TBL sessions on common immunological diseases on four different dates. 139 participants were divided into 4 groups [35 each in A, B, C, & 34 in D]. For TBL session 1, Group A and group B were the study and control groups, respectively. For the second session on different topics, the groups were reversed with group B and group A as the study and control groups, respectively. The same was followed for groups C and D. The means and standard deviations of the pre-test and post-test scores were compared after calculating the improvement in scores from pre- to post-tests. Repeated measures ANOVA suitably coded in SPSS for cross-over design was used to find out confounding by sequence of interventions with a p-value of <0.05 signifying the significance.  Students’ feedback on online TBL sessions was collected through a predesigned questionnaire on a 3-point Likert scale. The data were analyzed using SPSS version 22 and expressed as number and percentage.

**Results:**

The post-test scores of the students who participated in the TBL session were significantly higher when compared to the self-study (SS) arm. The overall improvement in scores was 4.98 (1.4) in TBL group, whereas in the SS arm it was only 2.29 (1.51). The new method was found far superior to the self-study method regardless of being applied before or after the comparison mode of self-study (P<0.0001). The scores of the self-study was marginally better when offered first rather than after a TBL session, indicating the negative effect of cross-over on SS mode (P=0.024). The overall response of our students toward the effectiveness of online TBL pedagogy was overwhelmingly positive in terms of an opinion survey which had a Cronbach’s alpha of 0.932. The majority (>80%) perceived TBL as an enjoyable active session that promoted their active participation and engagement through student-led discussions. Many stated that TBL enhanced their critical thinking, problem-solving ability, communication skills, and knowledge.

**Conclusion:**

TBL is an instructive and highly structured teaching-learning strategy, welcomed by the majority of our participants. Online TBL sessions are effective in fostering the students’ learning and can be used confidently when needed.

## Introduction

Over the last couple of decades, in majority of the medical schools worldwide, there was a paradigm shift in medical curricula from teacher-centered to learner-centered approach ( 1 - 2 ). Evidence suggests that teacher-centered approaches, where students are passive learners, are less appealing to the present millennial learners who are more digitally inclined and demand for more active and engaging learning environment ( 3 - 4 ). In contrast, student-focused active learning methods motivate learners, promote active participation, facilitate peer discussion, enhance communication, increase critical thinking and problem-solving ability, and thus foster knowledge retention ( 5 ). Rapid advancements in technology in recent years has led to the development and increased use of several student-focused, self-directed active learning approaches by medical educators ( 6 ). Problem-based learning (PBL), Patient-Oriented Problem Solving (POPS), Case-based learning (CBL), and Team-based learning (TBL) pedagogical strategies are some examples ( 7 - 10 ). Generally, the confidence of the students who learn through active learning modules is significantly high; thus, delivery of medical training through student-focused learning approaches improves medical education and student learning outcomes ( 11 ). 

Team-based learning is relatively a new, highly structured, and evidence-based collaborative active learning strategy, well suited for small group learning sessions ( 12 ). It is an instructor-led student-centered pedagogy delivered through several sequential phases. Students hold accountability for in-class and out-of-class preparation and master course concepts by working collaboratively with peers under the instructor’s frequent feedback ( 13 ). TBL is taught in three phases: pre-class preparation, in class readiness assurance testing, and knowledge application exercise ( 14 ). The pre-class preparation phase occurs before in-class TBL session. Students are required to study pre-reading material sent by the instructor in advance or attend flipped classroom lectures beforehand. This allows them to know the key learning issues and come prepared for the activity. In in-class TBL sessions, students are organized into a team of 5-6 members. At the beginning, students’ knowledge of the topic gained through pre-class preparation is tested by a set of questions (typically consisting of 10-20 questions; single best answer, true/false, multiple select type), wherein students complete the test individually (iRAT or individual Readiness assurance test) without referring to any study material or discussion with other students. After all students complete iRAT, they work together on the same set of questions in their team (tRAT: team readiness assurance test). During tRAT, students in the team are allowed to discuss the answers. Following completion of tRAT, the team must reach consensus after discussion with members and submit their collective answers that they think are correct. The instructor then provides immediate elaborative feedback on their answers and clears the concepts that students do not get or find difficult. The knowledge application exercise phase follows tRAT. In this phase, students work on a given challenging clinical case scenario and apply the conceptual knowledge (application exercise) that they have learnt in iRAT and tRAT to understand and digest various aspects of the clinical case such as pathogenesis, diagnosis, and management strategies, and others. The instructors provide elaborative feedback on case-related problems and additional explanation to students’ queries. Finally, to motivate students, best student and team is acknowledged by considering the performance in iRAT, tRAT, and application exercise ( 12 - 13 ).

Immunology is one of the basic science subjects included in the medical curricula. The course introduces medical students to various new terminologies and concepts pertaining to structure and function of the immune system, immunological diseases, pathogenic mechanisms, laboratory diagnosis, and management ( 14 - 15 ). Therefore, it is a challenge for medical students to learn and digest various concepts and correlate them to understand various aforesaid aspects of immunological diseases within a short period. Therefore, it is critical for immunology teachers to adopt a teaching method that promotes the students’ learning efficiently. Recently, at the College of Medicine and Health sciences (CoMHS), the curriculum was reviewed and reconstructed with an emphasis on active learning exercises. Team-Based Learning (TBL) method developed by Larry Michaelson in 1979 and used by medical educators across the globe was found to be suitable for small group session and was introduced into the immunology course ( 16 ). However, the present COVID-19 pandemic crisis resulted in closure of educational institutions. Continuation of education through virtual teaching by innovative learning and managing system became the need of the hour. Most of the educators have explored and found many effective e-teaching software to provide maximum possible benefits to students through online learning ( 17 ). Technology (InteDashboard^TM^ and OpenTBL^TM^) designed to support all key areas of online TBL sessions, namely iRAT, tRAT, and application exercise, are currently available ( 18 ). E-learning wave is a recent development, and students are in the process of adapting to new teaching and learning methods. Therefore, it is essential to know the opinion of students and explore their inclination toward novel e-learning teaching methodology ( 19 ). On thorough literature search, we found a few TBL studies in immunology conducted through face-to-face sessions at the institute, but there were no studies on online TBL method. Thus, the current study aimed at introducing online synchronous TBL pedagogy in immunology course and exploration of students’ perception on effectiveness of online TBL in promoting their learning skills. 

## Methods

The present crossover interventional study was conducted at College of Medicine and Health Sciences (CoMHS), Oman. The study was approved by the Institutional Research and Ethics Committee [Approval no: NU/COMHS/EBC0001/2021) and conducted after obtaining an informed consent from all the participants. 

### Study design

**Figure 1:**
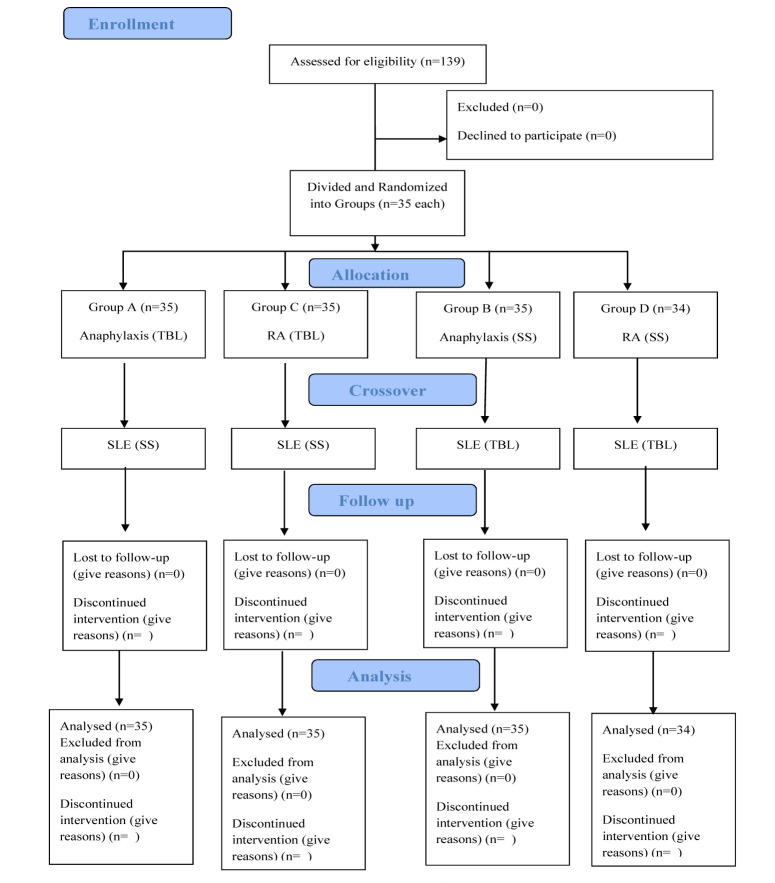
CONSORT 2010 Flow Diagram

TBL module used by several researchers was reviewed by subject experts, modified, and used in our study ( 8 , 13 , 14 ). The study design is depicted in Figure 1. MD4 medical students enrolled in medical course during the academic year 2020-21 were included in the study. Due to COVID-19 crisis and institutional lockdown, the TBL activity was conducted on Cisco Webex (purchased by our institution) online platform through breakout sessions. TBL sessions were conducted on common immunological diseases viz. systemic lupus erythematosus (SLE), Rheumatoid arthritis (RA), and Anaphylaxis. All students were informed about the activity two weeks prior to the session. The orientation sessions were given to overview the learning outcomes and objectives, information about learning platform and breakout rooms, and essentials of TBL process, team formations, team dynamics, team responsibilities, and grading (Fig 1). 

### Sample size

**Figure 2:**
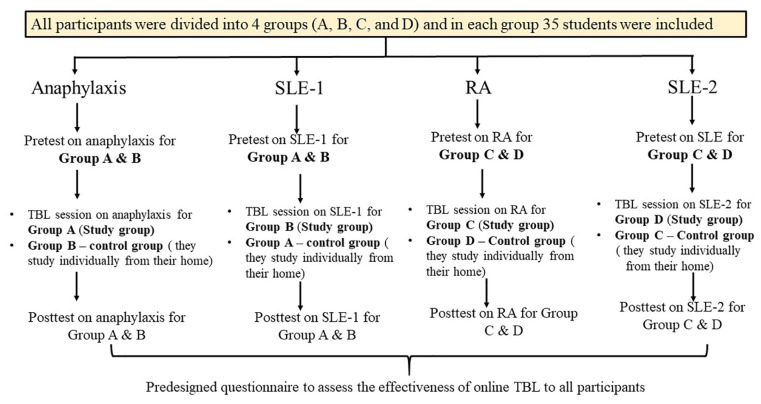
Study design of TBL activity. TBL: Team Based Learning

To find a difference of 3 in improvement of scores (post-pre) between TBL and control group with a pooled variance of 10, with a power of 80% and significance level of 5%, we found that the study needed a minimum sample size of 24 per group. The study was replicated four times to cover more participants as follows. The whole batch was divided into four groups: A, B, C, and D with 35 students in each, and each group was further divided into team of 6 members. TBL sessions on different topics for each group was conducted on prescheduled different dates and times. TBL sessions on Anaphylaxis, SLE-1, RA, and SLE-2 were scheduled for groups A, B, C, and D, respectively. During these TBL sessions, groups B, A, D, and C were considered as the control (self-study) groups, respectively (Fig 2). 

### Execution of the TBL sessions

**Figure 3:**
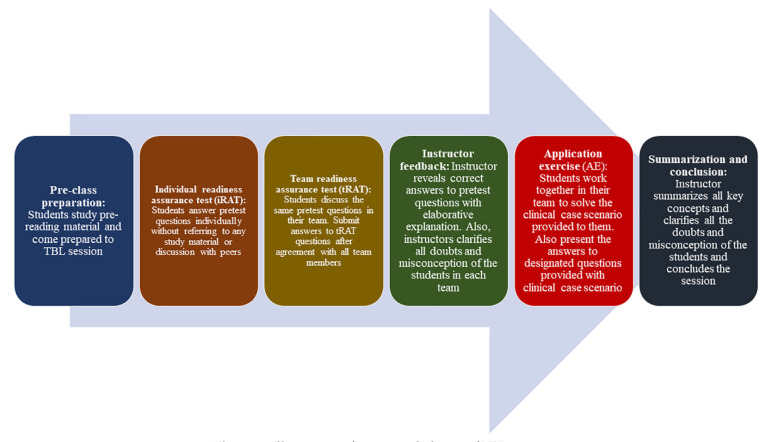
Illustration of sequential phases of TBL session. TBL: Team Based Learning

**TBL activity on anaphylaxis:** Students in groups A and B were enrolled in the study and control groups (self-study), respectively. The pre-class preparation study material with detailed information such as general characteristics, triggering/risk factors, pathophysiology, clinical features, complications, laboratory investigations, and management plan on Anaphylaxis was sent to all the students of group A and B through their institutional email one week prior to the activity. On the day of the session, 10 minutes prior to the beginning of the session, two separate Cisco-Webex links were sent to the students in groups A and B. All of them were first asked to complete the pretest questions on anaphylaxis (comprising of 10 questions; single best answer, true/false, and multiple select) sent by a google form to their institutional email within 15 minutes. Then, group B students (control group) were asked to do self-study individually on anaphylaxis at their home for 1 hour, while TBL session was conducted to group A students (study group). The group A students were divided randomly into teams comprising of 6 students in each team in Cisco-Webex break out rooms, and the TBL was conducted through standard sequential phases (Fig 3). 

The pretest completed earlier by the study group individually to know the knowledge acquired by studying pre-class reading material was considered as individual readiness assurance test (iRAT). Following iRAT, students were allowed to discuss the pretest questions in a team (team readiness assurance test; tRAT) for a period of 30 minutes. Upon completion, all teams were asked to submit their answers of tRAT questions after the collective decision of team members to the instructor’s email. Subsequently, the instructor gave feedback immediately to the individual team by revealing correct answer to each question with an elaborative explanation. Other doubts and misconceptions of each team were also clarified by the instructor. After completion of tRAT, all teams were asked to discuss (for half-hour) a clinical case scenario on anaphylaxis with a set of designated questions (application exercise) which was sent through their institutional email. In the final half hour time, all students were removed from breakout sessions and brought under a single Cisco Webex platform and were asked to present their answers to the set of questions given along with the clinical case scenario. Finally, the instructor provided answers and additional explanations to case-related students’ doubts and misconceptions. For promotion of the students’ participation and motivation, the best student and team was acknowledged by considering iRAT, tRAT, and application exercise performances. At the end of the session, post-test questions were sent through google survey form the link to all the group A members as well as group B members (who did self-study at home during TBL session of group A); they were asked to submit their answers within 15 minutes. All pre-test and post-test scores were collected for statistical analysis.

**TBL activity on SLE and RA:** Similarly, online synchronous TBL sessions for groups B, C, and D were carried out on separate pre-scheduled dates. Systemic lupus erythematosus topic was assigned and divided into two separate topics with different questions and clinical case scenario as SLE-1 and SLE-2. RA, SLE-1 and SLE-2 were the topics utilized for groups B, C, and D TBL sessions, respectively. Groups A, D, and C were the corresponding control groups for SLE-1, RA, and SLE-2 TBL sessions. At the end of each TBL session, pre-test and post-test answers of the study and control groups were collected and entered into Microsoft Excel sheet for statistical analysis.

Finally, after completion of all the TBL sessions, the pre-designed, self-administered questionnaire validated for its contents and relevance by Microbiology & Immunology, Medical education, and Medicine experts was used to get the feedback of the students regarding the effectiveness of online TBL activity in promoting their learning process. The link of the Google survey form of the questionnaire was sent to all the participants through their institutional email. The questionnaire was prepared on a 3-point Likert scale (agree, neutral, and disagree) on 12 items. All responses were collected for statistical analysis. 

### Statistical analysis

The students’ performances on pre-test and post-test were evaluated based on standardized answer keys. The improvement in scores following the sessions was calculated as dependent variables and these values were subjected to ANOVA using GLM: Repeated Measures in SPSS (statistical package for social sciences). The order in which the TBL and SS sessions were delivered was captured by a dummy variable using value 1 for SS-first and TBL-second, and 2 for TBL-first and SS-second. The model is set up as a repeated measures model defining a two-level within-subject factor (teaching mode). The results from the model are observed for the teaching mode and order in which the methods are delivered as explained earlier and a p-value for the F values are taken as significant if it is below 0.05. The profile plot is then used to find the ways in which these affect the improvement in scores.

Students’ perceptions regarding the new method of teaching were assessed using a questionnaire with items giving responses as three categories of disagree [1], neutral [2], and agree [3]. Responses were converted into percentages agreeing on each item on the questionnaire. The opinion questionnaire had a Cronbach’s alpha of 0.932, and all items were found to be valid and contributed equally to the overall opinion.

## Results

In total, 139 MD4 medical students of CoMHS of the academic year 2020-21 participated in the study. The cross-over nature of the educational intervention was tested for any interaction with the order as well as the teaching methods by repeated measures ANOVA. Table 1 shows improvements in post-test score from the pre-test score in various groups as per the order of delivery of teaching method. 

**Table 1:** Pre-test and post-test scores in various groups as per the order of delivery of the teaching method

Group		Order					
		TBL to SS		SS to TBL		Total	
		TBL	SS	TBL	SS	TBL	SS
A	Mean±SD	5.26±1.46	1.37±1.66			5.26±1.46	1.37±1.66
	N	35	35			35	35
B	Mean±SD.		4.91±1.09	2.77±1.52	4.91±1.09	2.77±1.52
	N			35	35	35	35
C	Mean±SD	4.94±1.75	2.77±1.21			4.94±1.75	2.77±1.21
	N	35	35			35	35
D	Mean±SD.		4.80±1.23	2.26±1.22	4.80±1.23	2.26±1.22
	N			35	35	35	35
Total	Mean±SD	5.10±1.61	2.07±1.61	4.86±1.16	2.51±1.39	4.98±1.40	2.29±1.51
	N	70	70	70	70	140	140

TBL and SS: F(1,137) = 321.309, p<0.0001, Period1 and Period2: F(1,137) = 5.236, p< 0.05

**Figure 4:**
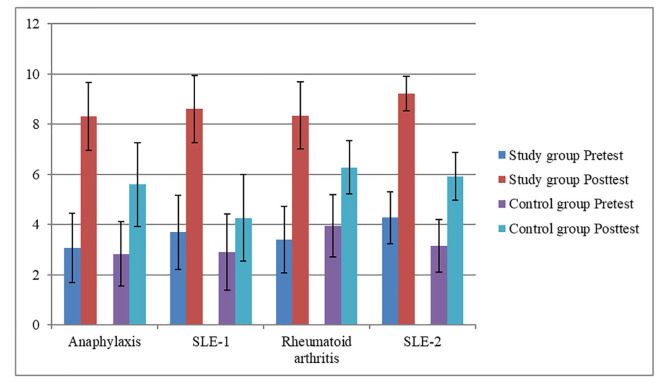
Pre-test and post-test scores of the study groups and controls

**Figure 5:**
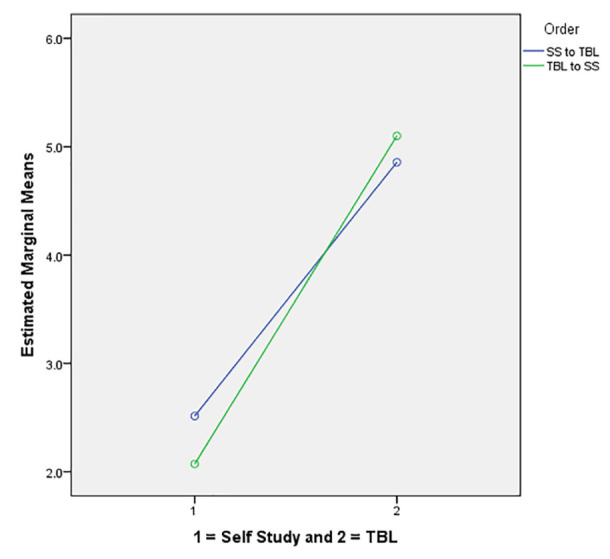
Scores of the Groups in TBL and SS arms TBL: Team Based Learning; SS arms: Self Study arms

The scores for TBL were consistently higher than the self-study (SS) across the groups A to D (Figure 4). As shown in the plot (Figure 5) for improvement of scores in TBL and SS arms, there were higher values for TBL (5.1±1.61 and 4.86±1.16) against (2.07±1.61 and 2.51±1.39) whichever order in which the method was delivered [F(1,137)=321.309, p<0.0001)]. The use of SS before TBL was found significantly better (2.51±1.39) than if it was delivered after TBL session [F(1,137)=5.236, p<0.05)]. The carryover effect of TBL to SS was negative, while SS had not much of a boosting effect on TBL if delivered one after the other.

Students’ feedback regarding their experience in TBL pedagogical teaching method are shown in Table 2. The majority (80%) of the students said TBL sessions were enjoyable. More than 85% stated TBL motivated them to engage in active discussion, enhanced their problem-solving skills, helped them to clarify their doubts and misconception, and improved their knowledge on the topic. About three-fourths said TBL facilitated critical thinking and motivation to learn better. More than 85% agreed that clinical case scenarios were interesting, and the facilitator provided useful and timely feedback and guided them in a right direction for active discussion. Nearly 91% said pre-class preparation material helped them to identify specific learning points which made them participate in discussion with peers actively and confidently during the TBL session. Furthermore, 88% opined iRAT and tRAT assisted their learning vastly. 

**Table 2:** Students’ perception on effectiveness of online TBL sessions

Questionnaire item	Disagree (%)	Neutral (%)	Agree (%)
I enjoyed the TBL session.	5 (3.7)	23 (16.8)	109 (79.6)
TBL motivated me to engage in active discussion.	3 (2.2)	11 (8.1)	118 (88.1)
TBL enhanced my communication skills with peers.	11 (8.2)	21 (15.6)	103 (76.3)
TBL session increased my problem-solving ability.	2 (1.5)	12 (8.8)	122 (89.7)
Facilitator helped to focus discussion and learning in right direction.	2 (1.5)	14 (10.3)	120 (88.2)
Clinical case scenarios were interesting and facilitated active discussion.	5 (3.7)	11 (8.1)	120 (88.2)
TBL enhanced my critical thinking to solve clinical problems.	4 (2.9)	26 (19.1)	106 (77.9)
TBL increased my ability to apply and correlate accumulated knowledge to solve the clinical case.	5 (3.7)	19 (14.0)	112 (82.3)
Facilitator gave useful and timely feedback on key aspects.	5 (3.7)	15 (11.0)	116 (85.3)
TBL activity was helpful in clarifying my doubts and misconceptions related to the key concepts of the topic.	1 (0.7)	13 (9.6)	122 (89.7)
Pre-reading material helped me to identify key learning points for discussion during the session	3 (2.2)	9 (6.7)	123 (91.1)
Individual and team readiness assurance tests at the beginning of the session assisted my learning.	4 (3.0)	12 (9.0)	117 (88.0)
TBL session improved my overall knowledge on the topic.	4 (3.0)	11 (8.2)	120 (89.0)

TBL: Team Based Learning

## Discussion

The conventional face-to-face TBL is a relatively new pedagogical learning approach introduced by many medical educators globally ( 20 , 21 ). The ongoing COVID-19 pandemic resulted in institutional lockdown and paved the way for online mode of teaching to continue the students’ learning. This paradigm shift in education hampered holding small group sessions including conventional TBL. However, development of several web-based techniques in teaching-learning made it possible to continue small group sessions through virtual online platforms ( 19 ). The current study sought to explore medical students’ perception on online synchronous TBL sessions conducted on Cisco Webex online platform in promoting their learning process in the Immunology course. 

Results of our study indicated that students’ perception on online TBL sessions was overwhelmingly positive. One of the advantages of TBL is smaller group size comprising of five or six students compared to 10-15 in PBL. This enhances the students’ active participation in discussion and peer learning. Furthermore, evidence suggest that pre-readiness before the session, readiness assurance process during the session, initially individual test followed by team test, and immediate feedback from the expert instructor increase motivation and engagement among students in the learning process. In contrast, PBL sessions facilitated by instructors with variable experience (need not be subject experts), limited direction and lack of immediate instructor’s feedback, and large group size may hinder the learning process of the students ( 11 ). Cisco Webex online tool has an option namely breakout rooms in which students can be divided into small teams and the instructor can monitor one team at a time. Also, we successfully carried out the sequential phases of conventional TBL such as iRAT, tRAT, application exercise, and instructor feedback in Cisco Webex breakout rooms. Most of our participants (88%) reported that TBL had increased their motivation and engagement in active discussion. Collaboration and excellence in communication are two essential prerequisites for medical professionals to work efficiently in complex health care system ( 22 ). The structured TBL favors students to work in a group, communicate with each other, and prove/disprove a statement in a healthy conversation to arrive at a correct answer. Notably, three fourths of our participants agreed that TBL sessions improved their communication skills, collaborative learning, critical thinking, and problem-solving ability. One of the advantages of TBL is that students come prepared by studying specified pre-reading material before the session. This allows them to have sufficient requisite knowledge of the topic prior to the session, thus promoting their active discussion in the right direction during the session to acquire essential knowledge ( 14 ). Evidence suggests the quality of in-class group discussion improves when students attend session with a prior designated preparation ( 13 ). Most of our participants (91%) opined that pre-reading helped them to identify specific learning issues, which enabled them to engage in active discussion confidently during the TBL session. Additionally, individual and team readiness assurance tests at the beginning of the session followed by immediate elaborative feedback on difficult questions/content of the topic help the students to correct their mistakes and gain the right knowledge. The iRAT and tRAT allow the instructor to immediately assess the learners’ knowledge and understanding, thereby addressing their specific needs ( 23 ). More than 85% of our students expressed that readiness assurance tests (iRAT and tRAT), facilitator’s immediate feedback, and appropriate guidance assisted their learning. Team-based learning moves beyond just acquisition of knowledge by emphasizing application of knowledge in solving real-life case scenarios through group discussion ( 24 ). Constructing a relevant and authentic clinical scenario is an essential requisite to foster reasoning and problem-solving skills of the students ( 25 ). In the same line, the majority of our students said the clinical case-scenarios were relevant and interesting. Case scenarios facilitated their active discussion and application of knowledge that had been learnt through readiness assurance tests and immediate feedback to resolve the cases. Additionally, clarification of the students’ doubt and elaborative explanation to case-related problems by the instructor enhance the acquisition and retention of correct knowledge by students. Evidence suggests student-focused active learning strategies where students learn by active participation and group discussion improves their knowledge ( 26 ). As a support of this finding, - scores of our students who learnt through TBL session were significantly high compared to those who did self-study at their residence. However, we could not monitor the self-study group. 

Finally, though online TBL sessions are found to be effective, we realized several barriers that need to be considered. First of all, continuous power supply and good Internet connectivity are essential requisites, without which online TBL will be a failure. Secondly, in conventional face-to-face TBL activity, all students are involved in the activity under one roof, so that all can be monitored continuously, and individual and team members’ doubt clarification and feedback by the instructor will reach the whole group simultaneously, which in turn enhances the students’ motivation and learning. In contrast, in online sessions, students are divided into teams through breakout session and there will be no contact between different teams. This might be a hindrance to students’ learning process. Thirdly, facilitator cannot monitor all the teams simultaneously as he must move online from one team to another team; thus, it is difficult to monitor the discussions all the time. Additionally, feedbacks need to be given separately to individual teams; thus, it necessitates the involvement of more instructors. Fourthly, to ensure optimal students’ learning environment, instructors must possess the sound knowledge regarding the working principles of the software. Lastly, institutional support to purchase appropriate software and optimal organization and support is vital ( 27 ). 

### Limitation

Our online TBL study had few limitations. First, we could not monitor all students’ discussions in all the breakout rooms simultaneously as the facilitator needed to switch from one breakout room to another. Secondly, pre-test and post-tests are sent through online google forms; hence, it was difficult to know whether answers were shared by the students. Lastly, it is a single centered study with a small sample size; hence, the results of the study cannot be generalized.

## Conclusion

Institutional lockdown or restriction due to COVID-19 crisis should not be a hindrance to conducting student-focused active learning programs. Our study results showed TBL pedagogy conducted through virtual online platform had equal potents to foster the students’ motivation, engagement, and knowledge acquisition. Application of the basic knowledge and what had been learnt through sequential phases of TBL sessions improves the learners’ high order cognition and learning outcomes. Therefore, live online synchronous TBL sessions could be used confidently when situation demands to assist the students to prepare well to meet the demands of the increasing complex health care systems in which they will work. 

## Acknowledgement

We are deeply indebted to all MD 4-year medical students of academic year 2020-21 of College of Medicine and Health Sciences, National University of Science and Technology (NUST) for their active participation and helping us to complete the study. We would also like to thank Ms. Winnie Philip, Lecturer in Biostatistics, Research Unit, College of Applied Medical Sciences, King Saud Bin Abdul-Aziz University for Health Sciences, King Abdullah International Medical Research Centre, NGHA, Riyadh, KSA Riyadh, KSA for helping us perform the statistical analysis for cross over trials.

**Conflict of Interest:** None Declared. 
